# They’re heating up: Internet search query trends reveal significant public interest in heat-not-burn tobacco products

**DOI:** 10.1371/journal.pone.0185735

**Published:** 2017-10-11

**Authors:** Theodore L. Caputi, Eric Leas, Mark Dredze, Joanna E. Cohen, John W. Ayers

**Affiliations:** 1 The Wharton School, University of Pennsylvania, Philadelphia, PA, United States of America; 2 Department of Epidemiology and Public Health, University College Cork, Cork, Republic of Ireland; 3 Drug Policy Institute, University of Florida College of Medicine, Gainesville, FL, United States of America; 4 Stanford Prevention Research Center, Stanford University School of Medicine, Palo Alto, CA, United States of America; 5 Department of Computer Science, Johns Hopkins University, Baltimore, MD, United States of America; 6 Institute for Global Tobacco Control, Bloomberg School of Public Health, Johns Hopkins University, Baltimore, MD, United States of America; 7 Graduate School of Public Health, San Diego State University, San Diego, CA, United States of America; University of California, San Diego, UNITED STATES

## Abstract

Heat-not-burn tobacco products, battery powered devices that heat leaf tobacco to approximately 500 degrees Fahrenheit to produce an inhalable aerosol, are being introduced in markets around the world. Japan, where manufacturers have marketed several heat-not-burn brands since 2014, has been the focal national test market, with the intention of developing global marketing strategies. We used Google search query data to estimate, for the first time, the scale and growth potential of heat-not-burn tobacco products. Average monthly searches for heat-not-burn products rose 1,426% (95%CI: 746,3574) between their first (2015) and second (2016) complete years on the market and an additional 100% (95%CI: 60, 173) between the products second (2016) and third years on the market (Jan-Sep 2017). There are now between 5.9 and 7.5 million heat-not-burn related Google searches in Japan each month based on September 2017 estimates. Moreover, forecasts relying on the historical trends suggest heat-not-burn searches will increase an additional 32% (95%CI: -4 to 79) during 2018, compared to current estimates for 2017 (Jan-Sep), with continued growth thereafter expected. Contrasting heat-not-burn’s rise in Japan to electronic cigarettes’ rise in the United States we find searches for heat-not-burn eclipsed electronic cigarette searches during April 2016. Moreover, the change in average monthly queries for heat-not-burn in Japan between 2015 and 2017 was 399 (95% CI: 184, 1490) times larger than the change in average monthly queries for electronic cigarettes in the Unites States over the same time period, increasing by 2,956% (95% CI: 1729, 7304) compared to only 7% (95% CI: 3,13). Our findings are a clarion call for tobacco control leaders to ready themselves as heat-not-burn tobacco products will likely garner substantial interest as they are introduced into new markets. Public health practitioners should expand heat-not-burn tobacco product surveillance, adjust existing tobacco control strategies to account for heat-not-burn tobacco products, and preemptively study the health risks/benefits, popular perceptions, and health messaging around heat-not-burn tobacco products.

## Introduction

The tobacco control community is still deciding how to address the unexpected rise in popularity of electronic cigarettes [1–2]. However, there is another product innovation already emerging: Heat-not-burn tobacco products.

Battery powered devices that heat leaf tobacco to approximately 500 degrees Fahrenheit to produce an inhalable aerosol, heat-not-burn tobacco products are being introduced in markets around the world by tobacco companies seeking to appeal to trendy or potentially health-conscious consumers who still demand the “throat-hit” delivered by combustible cigarettes but not by electronic cigarettes [3]. Japan has been the focal market to test the potential of heat-not-burn as a cigarette alternative, where manufacturers have marketed several heat-not-burn brands nationwide, including Japan Tobacco’s “Ploom TECH” device in March 2016, Philip Morris International’s “iQOS” (or “ICOS”) device in April 2016, and British American Tobacco’s “Glo” device in December 2016 [4]. Tobacco industry leaders have predicted heat-not-burn products are poised to further displace traditional cigarette smoking and, by extension, tobacco control strategies typically framed around cigarettes. Yet, little is known about the popularity of these products. For the first time, we describe trends in the popularity of heat-not-burn tobacco products in their Japanese test market and compare these trends with historical trends for electronic cigarettes to understand the growth potential of this new product globally.

## Materials and methods

Traditional survey-based assessments are not available to describe the heat-not-burn tobacco market because survey data are delayed or simply fail to ask about heat-not-burn tobacco. However, examining how individuals search online takes surveillance to the next level by revealing both the searcher’s thoughts, through the types of queries undertaken, and their actions toward product use, through engaging in the search behavior itself, without any instrumental priming [5,6]. Search query trends were used to first describe the emergence of electronic cigarettes by Ayers and colleagues in 2011 [1] when the tobacco control community was focused on a snus pandemic that never materialized. Moreover, search query trends consistently foreshadow consumer behaviors outside tobacco, including public adoption of novel products [7].

Monthly Google query trends were monitored from January 1, 2010 through September 13, 2017 (google.com/trends). All queries including “heat-not-burn” and/or the most popular brands [8] originating from Japan, as well as their Japanese translations were monitored in aggregate representing heat-not-burn popularity. Specifically, we used www.tobaccowatcher.org to search news archives for stories on heat-not-burn tobacco products to identify the common Japanese nomenclature around these products, discovering 加熱式たばこ and 加熱式タバコ were the most common references to generic heat-not-burn tobacco products. We also identified all brands currently discussed in the media using the same strategy, including “iqos”/”icos” [アイコス], “plume” [プルーム], and “glo” [グロー].

Given heat-not-burn tobacco products are not marketed nationally outside of Japan we compared Japanese heat-not-burn search trends against searches for electronic cigarettes in the United States. This analysis was intended to compare the growth rate and growth potential of heat-not-burn against the past growth of electronic cigarettes using a common metric: Internet search. Moreover, because nicotine containing electronic cigarettes are not (legally) sold in Japan we could not validly compare Japanese heat-not-burn searches against electronic cigarettes within Japan. All queries including the terms “electronic cigarette(s),” “ecig(s),” “e-cig(s),” “e cig(s),” “e cigarette(s),” “e-cigarette(s),” “vape(s),” “vaper(s),” or “vaping” were monitored in aggregate alongside the names of the most popular e-cigarette brands “blu,” “eleaf,” “eversmoke,” “greensmoke,” “green smoke,” “halo cigs,” “halo ecigs,” “njoy,” “v2,” “vuse,” “el ray,” “vapestick,” “21st century smoke,” “vaporx,” “markten,” “v2pro,” or “vapor couture” representing electronic cigarettes popularity building on past work [2,9]. Because all searches are typically increasing, a normalized, monthly ratio of focal queries to all queries was analyzed (relative search volume [RSV]), alongside raw volume estimates computed by multiplying the query fraction and the estimated number of total searches from comScore.

We evaluated changes in average monthly heat-not-burn queries in Japan and electronic cigarette queries in the United States using relative differences of means with confidence intervals calculated via bootstrap resampling. To make predictions through 2018 we used Hyndman and Khandakar’s autoregressive integrated moving average algorithm that uses historical data to predict future values [10]. Ninety-five percent prediction intervals were generated from 1000 simulations of the ARIMA model with resampling errors from the fitted model (rather than normal errors). Predictions for 2018 were compared to the monthly average queries in 2017. All analyses were performed using R ver 3.4.3.

## Results

**Fig 1:**
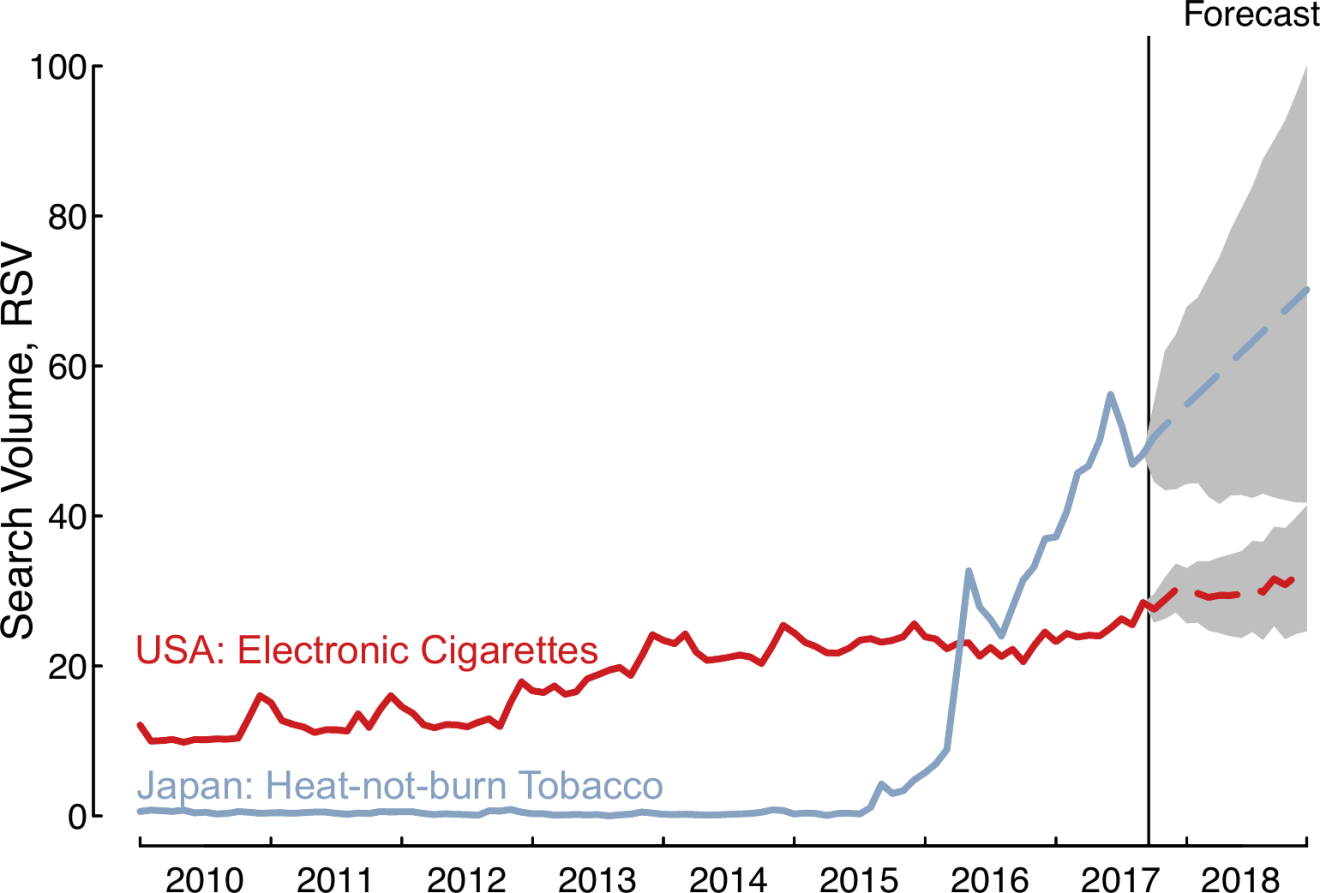
Google searches for heat-not-burn tobacco outpace past rise of electronic cigarettes. The above figure shows the Relative Search Volume (scaled from 0–100 and adjusted for number of total Google search volumes per month in Japan and the USA) for heat-not-burn and electronic cigarette products.

Heat-not-burn searches originating in Japan have experienced tremendous growth (**Fig 1**). Since the introduction of Philip Morris International’s iQOS brand in select Japanese cities in November 2014, searches for heat-not-burn products have increased substantially. Average monthly searches rose 1,426% (95%CI: 746–3,574) between the first (2015) and second (2016) complete years heat-not-burn tobacco was marketed. Queries for heat-not-burn products continued to grow an additional 100% (95%CI: 60–173) between the products second (2016) and third years on the market (Jan-Sep 2017). In practical terms, there are now between 5.9 and 7.5 million heat-not-burn related Google searches in Japan each month based on the latest search estimates for September 2017. Moreover, forecasts relying on the historical trend suggest heat-not-burn searches will increase an additional 32% (95%CI: -4 to 79) during 2018, compared to current estimates for 2017 (Jan-Sep), with further growth expected.

Queries for heat-not-burn in Japan occur more frequently than queries for electronic cigarettes in the United States, with the Japanese heat-not-burn queries first eclipsing electronic cigarette queries in April 2016. Further, the change in average monthly queries for heat-not-burn in Japan between 2015 and 2017 was 399 (95% CI: 184–1,490) times larger than the change in average monthly queries for electronic cigarettes in the Unites States over the same time period, increasing by 2,956% (95%CI: 1,729–7,304) compared to only 7% (95%CI: 3–13). These results indicate that interest in heat-not-burn may outpace interest in electronic cigarettes (in areas where they are introduced) not just now but for years to come.

## Discussion

Millions are seeking out heat-not-burn tobacco products in Japan each month, with growth rates and projections for future growth eclipsing the past rise of electronic cigarettes. These findings suggest the heat-not-burn market is poised for explosive global growth.

Given these products’ success in Japan, it is unsurprising that the heat-not-burn market is expanding. Heat-not-burn tobacco products have recently been released in metropolitan test markets in more than twenty nations, including the United Kingdom, Russia, and Korea [11]. Moreover, Philip Morris International’s iQOS recently entered the Food and Drug Administration approval process to begin sales in the United States [12]. Our findings suggest that tobacco control leaders should prepare for substantial demand for these products when they are introduced to new markets and/or expanded in existing markets. Traditional surveillance strategies to monitor these markets must be developed and put in place now [13], including further analyses of other big media data [14]. In particular, while our study identified a growing aggregate trend, use of specific products or types of products must be monitored, along with variations in heat-not-burn tobacco product receptivity across demographic groups.

Demand for heat-not-burn tobacco products presents a host of tobacco control challenges similar to electronic cigarettes and new challenges specific to these products [15]. Heat-not-burn tobacco products have been advertised as reduced-risk tobacco products in their Japanese test market [16], and these marketing messages will undoubtedly contaminate other markets even where such messaging is banned. Tobacco control advocates will have to develop strategies to both discover and then disseminate messages about the health risks associated with these products. Further, tobacco control advocates will have to consider making adjustments to existing tobacco control policies so that they apply to heat-not-burn tobacco products. For example, policy makers want to extend existing indoor smoking bans to include the emissions from heat-not-burn tobacco products, whether to stigmatize their use or protect bystanders from potentially hazardous emissions [17].

The Japanese test market may not effectively translate to other markets for two principal reasons. First, novel, tech-related product adoption is potentially more common in Asian societies; however, a perpetually weak Japanese economy may be reducing this potential bias where cost is a significant barrier regardless of the public’s inclination [4]. Second, nicotine-infused liquid, such as those used in electronic cigarettes, are strictly regulated as pharmaceutical products, while pipe tobacco used in heat-not-burn tobacco products are not [18]. Therefore, the Japanese heat-not-burn market may be advantaged by being the only non-combustible novel alternative to cigarette smoking. Last, some may question the use of search query trends, however, these data presaged the global rise of electronic cigarettes [1–2], have produced actionable insights across tobacco control that presage traditional data or fill data gaps [19–22], and search query trends for tobacco have been validated against survey based criterion [23].

Our findings are a clarion call to action. As of July 18, 2017 there are fewer than 26 studies to date including “heat not burn” or “heat-not-burn” in the title or Abstract on PubMed [24–28]. Only 1 study aimed to describe use of the product in Japan and that study was before heat-not-burn tobacco was launched nationwide [8]. Other studies are mostly laboratory-based product descriptions or speculative opinion pieces. Health agencies, policy-makers and health practitioners should fill this gap and preempt rising popularity of these products by investing in tobacco control research and strategies for heat-not-burn tobacco products now.
